# Molecular identification of *Entamoeba* spp. in humans and cattle in Baghdad, Iraq

**DOI:** 10.14202/vetworld.2024.1348-1355

**Published:** 2024-06-21

**Authors:** Sahad M. K. Al-Dabbagh, Haider H. Alseady, Enas J. Alhadad

**Affiliations:** 1 Department of Medical Laboratory Techniques, Institute of Medical Technology Al-Mansour, Middle Technical University, Baghdad, Iraq; 2 Department of Medical Laboratory Techniques, Technical Institute of Babylon, Al-Furat Al-Awsat Technical University, Babylon, Iraq

**Keywords:** cattle, *Entamoeba* spp, human, phylogenetic, sequence analyses

## Abstract

**Background and Aim:**

A total of 10% of the global population succumbs to amoebiasis yearly, equating to 50,000–100,000 recorded fatalities. It is closely associated with contaminated food and water supplies due to human feces. The disease’s pathophysiology remains a subject of ongoing debate among experts. Some experts attribute the role of the host’s conditions, parasite species and strain, and infection intensity in eliciting clinical symptoms. The aim of this study was to perform molecular identification of *Entamoeba* species isolated from humans and cattle.

**Materials and Methods:**

Stool samples from three hundred patients and one hundred cattle were collected from different regions, age groups, and sexes in Baghdad for microscopic examination. One hundred randomly chosen patient and cattle stool samples underwent microscopic examination and conventional polymerase chain reaction (PCR) targeting the 18S rRNA gene. Phylogenetic tree analyses were performed for *Entamoeba* species identification.

**Results:**

The infection rate in humans differed significantly (p < 0.05) between age groups and genders, totaling 38%. The infection rate in cattle, determined by conventional PCR, differed significantly (p < 0.05) between age groups and genders, amounting to 58%. Ten PCR products with positive results were sequenced and deposited in GenBank database. Sequence analysis detected that Eight sequences belong to *E. histolytica* (OM268853.1, OM268854.1, OM268855.1, OM268857.1, OM268858.1, OM268860.1, OM268861.1, and OM268862.1) and two sequences belong to *E. dispar* (OM268856.1 and OM268859.1) in humans, while 10 sequences (ON724165.1 to ON724174.1) belongs to *E. histolytica* in cattle.

**Conclusion:**

The increased susceptibility of cattle to *E. histolytica* suggests a considerable role in human infection and substantial public health risks. Further research should be conducted on the many virulence factors such as HM1:IMSS strain, cysteinprotease (Cp1), Gal/lectin, etc. of *E. histolytica* and *E. dispar*.

## Introduction

Around the world, 50 million cases of amebiasis lead to 40,000–100,000 annual fatalities [1]. *Entamoeba histolytica* is a unicellular protozoon identified by pseudopodia and noted as the third deadly parasitic origin after Schistosomiasis and Malaria [2]. *E. histolytica* multiplication in the gastrointestinal tract produces cysts, which then progress out with stool and transmission to other healthy persons after consumption of contaminated food and water, causing ulceration and dysentery with bloody diarrhea in some cases and spreading to extraintestinal sites to produce abscesses of the liver, weight loss, colitis, and abdominal pain [2, 3]. *E. histolytica* employs toxins, pores, and adhesions as its primary virulence factors, resulting in tissue damage [2]. In laboratory animals, *Entamoeba dispar* can induce severe intestinal damage [4]. It is considered a chronic commensurable in humans, nevertheless, with no pathogenic traits, producing an asymptomatic carrier state and often being far more prevalent globally than *E. histolytica* [5]. The liver and intestines may suffer damage [6]. Poor sanitation, environmental pollution, overpopulation, inadequate education, and contaminated food and water promote *E. histolytica* transmission [7]. Studies have indicated a higher incidence of *E. histolytica* infection compared to *E. dispar* infection [8–10]. While other studies confirm a higher infection rate for *E. discar* than *E. histolytica*, Al-Hilfi *et al*. [11] present the opposite finding. The genetic diversity of *Entamoeba* species has received little research attention in Iraq [2, 12, 13]. Alarady and Jasim [14] found that *E. dispar* in cows and sheep was 35.7% and 21.45%, while *E. histolytica* was 85.7% and 21.4%, respectively, in Iraq.

Therefore, this study aimed to perform genomic species analyses in *Entamoeba* species isolated from humans and cattle and identify the associated risk factors of age and gender on the infection rates to detect the true pathogenicity.

## Materials and Methods

### Ethical approval and Informed consent

Ethical approval was obtained from the guidance of Research, Publication, and Ethics of the College of Veterinary Medicine, University of Baghdad, Iraq (No. BMS/0231/016), which complies with all relevant Iraqi laws. A verbal consent form was obtained from the animal’s owners. The families of study participants and hospital management granted consent. Families were informed orally about the study’s aim to facilitate sampling from hospitals and healthcare facilities.

### Study period and location

The study was conducted from September 01, 2021, to March 2022, at the Laboratory of Medical Laboratory Techniques, Institute of Medical Technology Al-Mansour, middle Technical University.

### Samples collection

Three hundred patients (consisting of 187 females and 113 males, aged from <10 to more than 40 years) provided 25 g stool samples from Al-Zahra Teaching Hospital (80), Ibn -Al-Baladi Hospital (100), and Al-Kadhimya Hospital (120) in Baghdad province. One hundred cattle stool samples, <1 year to over 3 years in age and comprising 56 females and 44 males, were gathered from Abu-Ghraib (25), Al-Mahmodeyia (25), Al-Shualah (25), and Al-Nahrwan regions (25). In cold bags, clean plastic containers containing stool samples, each with a sequential number and a pair of disposable gloves were transported to the laboratory. The data included sex and age information for all samples. For microscopic diagnosis of *Entamoeba* spp., 15–20 g of each collected stool sample was preserved in 10% formalin and stained with Lugol’s iodine (1%) and 0.9% saline, while 1–2 g was used for further tests such as ELISA and molecular [15].

### DNA extraction

The DNA in stool samples was extracted using a Bioneer (Korea) kit. The DNA was extracted using a stool lysis protocol with proteinase K, following the manufacturer’s instructions. The nanodrop spectrophotometer (Thermo Fisher, USA) was used to check the genomic DNA extraction, which was then stored at −20°C.

### Polymerase chain reaction (PCR)

This assay of PCR was accomplished for specific primers for identified *Entamoeba* spp. depend on 18S rRNA gene, the primers were designed based on National Center for Biotechnology Information (NCBI) GenBank established sequence of *Entamoeba* spp. RL2 partial 18S rRNA gene (GenBank: FR: 686362.1) by utilizing NCBI GenBank database and primers 3 plus online (Bioneer). These primers were used to amplify 590 bp of the 18S rRNA gene in *Entamoeba* spp. 18S rRNA –F primers (ATTGGAGGGCAAGTCTGGTG) and 18S rRNA-R primers (CATACTCCC CCTGAAGTCCA). Thus, the PCR master mix was accomplished using the (AccuPower® PCR Premix kit, Bioneer). The PCR premix tube included freeze-dried pellets of Taq DNA polymerase IU, tris HCL (PH 9.0) 10 Mm, dNTPS 250 μm, Mg CL21.5 Mm, KCL 30 Mm, Tracking dey and stabilizers), and the polymer chain reaction master mix was achieved based on kit instructions in 20 μL total volume by adding 1 μL of 10 pmole of forward primers and 1 μL of 10 pmole of reversed primers and 5 μL of purified g DNA, then added deionizer premix by PCR water to increase volume to 20 µL and mixing with Exispin vortex centrifugation (Bioneer). The reaction was accomplished in a thermocycler (Mygene, Bioneer) as follows: The reaction undergoes initial denaturation for 5 min at 95°C, followed by 30 cycles then 95°C denaturation cycles of 30 s each, annealing cycles of 30 s each at 58°C, followed by extension cycles of 1 min each at 72°C, and ended with a final extension of 5 min at 72°C. 1% agarose gel electrophoresis and ethidium bromide staining under ultraviolet light were used to test the PCR products.

### DNA sequence methods

Identification of *Entamoeba* spp. was based on phylogenetic tree analysis of the 18S rRNA gene sequences. The 18S rRNA gene (590 bp PCR product) was purified using an EZEZ-10 spin column and then sent to Bioneer in Korea for DNA sequencing with 18S rRNA forward primers and AB DNA sequence system. Phylogenetic analyses were carried out based on NCBI Basic Local Alignment Search Tool (BLAST) alignment results and the Neighbor Distance method in Mega version 6 (https://www.megasoftware.net).

### Statistical analysis

The data’s values are presented in both percentage and numerical form. Chi-square test was conducted to analyze the percentage discrepancies using Statistical Package for the Social Sciences Statistics 22 software (IBM Corp., NY, USA). A p < 0.05 was considered statistically significant [16].

## Results

**Figure-1:**
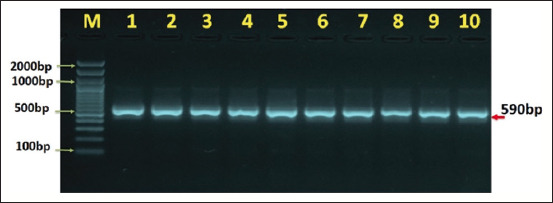
Agarose gelatin electrophoresis demonstrating the analysis of PCR products for the 18S ribosomal RNA gene of *Entamoeba* species and human feces samples, where M (marker) is a positive PCR result for the 18S ribosomal RNA gene at (590 base pair) and a marker (2000–100 base pair).

**Figure-2:**
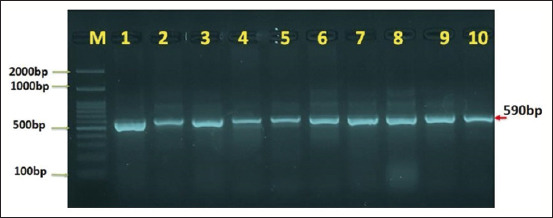
Agarose gelatin electrophoresis demonstrating the analysis of PCR products for the 18S ribosomal RNA gene of *Entamoeba* species and samples of local cattle feces, where M (marker) is a positive PCR result for the 18S ribosomal RNA gene at (590 base pair) and a marker (2000–100 base pair).

18s ribosomal RNA gene primers were used in PCR to distinguish *Entamoeba* species from human and cattle stool samples. Results from agarose gel electrophoresis demonstrated distinguishable bands at 590 pb for *Entamoeba* species in human and cattle stool samples (Figures-1 and 2).

Thirty-eight out of 100 tested samples were positive using conventional PCR, yielding a 38% infection rate in humans. Age groups <10 years and ≥40 years had 65% and 55% infection rate, respectively (), followed by age groups 20–30 years (40%), compared to the lowest (10%) in age groups 30–40 years with a significant difference at p < 0.05 (Table-1). About 52% of females had the infection compared to 24% of males, with a statistically significant difference at p < 0.05 (Table-2).

**Table-1:** Total infection rates of *Entamoeba* spp. in humans according to age groups by conventional PCR.

Age/years	No. of samples examined	No. of positive samples	Percentage
<10 years	20	13	65
10–20 years	20	4	20
20–30 years	20	8	40
30–40 years	20	2	10
≥40 years	20	11	55
Total	100	38	38
Chi-square 18.8, p = *0.001 (HS)			

* HS=Highly significant difference at p < 0.05. PCR=Polymerase chain reaction

**Table-2:** Total infection rates of *Entamoeba* spp. in humans according to gender by conventional PCR.

Sex	No. of samples examined	No. of positive samples	Percentage
Females	50	26	52
Males	50	12	24
Total	100	38	38
Chi-square 8.31, p = *0.004 (HS)			

* HS=Highly significant difference at p < 0.05. PCR=Polymerase chain reaction

**Figure-3:**
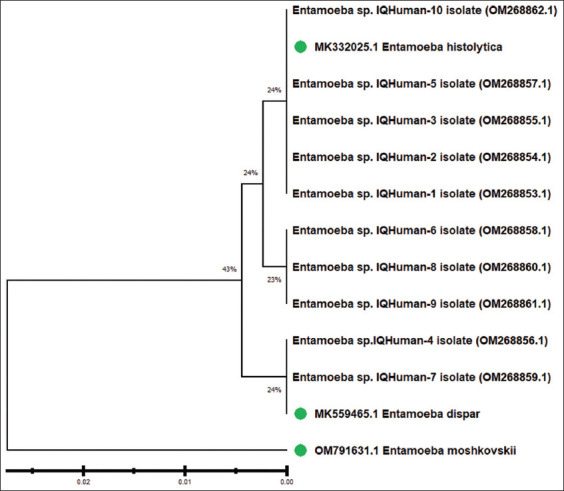
Isolated human *Entamoeba* spp. was employed in phylogenetic tree analysis dependent on the partial sequencing of the 18S ribosomal RNA gene using genomic species typing analyses. The Unweighted Pair Group Methods with Arithmetic Mean (UPGMA tree) were used to construct the phylogenetic tree (MEGA 6.0 version). The isolated human *Entamoeba* spp. (No. 1, No. 2, No. 3, No. 5, No. 6, No. 8, No. 9, and No. 10) were identified as being closely linked to the *E. histolytica* (MK332025.1) strain by NCBI-BLAST. 4 and No. 7) were identified as being closely linked to the *E. dispar* (MK2559465.1) strain by NCBI-BLAST, with total genomic alterations of 0.02%–0.01%.

DNA sequence in humans 18S ribosomal (RNA) gene sequencing was performed on isolated *Entamoeba* species and related *Entamoeba* spp. from NCBI-GenBank. Humans appeared closely related to *E. histolytica* (MK332025.1) in the phylogenetic tree analysis of genomic relationships with *Entamoeba* species (No.1, NO.2, NO.3, NO.5, NO.6, NO.8, NO.9, and No.10). Humans were found to be closely related to *E. dispar* (MK559465.1) according to NCBI-BLAST analysis with *Entamoeba* species No.4 and No.7. Isolated human *Entamoeba* species (No.1, NO.2, NO.3, NO.5, NO.6, NO.8, NO.9, and No. 10) demonstrated genomic homology ranging from 99.09% to 100% with the *E. histolytica* reference sequence MK332025. No.4 and No.7 *Entamoeba* species show 100% genomic homology with NCBI-BLAST *E. dispar* (MK559465.1). The human *Entamoeba* spp. isolates submitted to NCBI GenBank with accessions OM268853.1 to OM268862.1 (Figure-3 and Table-3) were verified.

**Table-3:** The NCBI-BLAST homologous sequencing identity percentage between isolated human *Entamoeba* spp. and NCBI-BLAST closed genomic relationship the isolated *Entamoeba* species.

*Entamoeba* spp. isolate	Accession number	Homology sequence identity (%)		

		Identical *Entamoeba*	Accession number	Identity (%)
IQ-Human No. 1	OM268853.1	*Entamoeba histolytica*	MK332025.1	100
IQ-Human No. 2	OM268854.1	*Entamoeba histolytica*	MK332025.1	100
IQ-Human No. 3	OM268855.1	*Entamoeba histolytica*	MK332025.1	100
IQ-Human No. 4	OM268856.1	*Entamoeba dispar*	MK559465.1	100
IQ-Human No. 5	OM268857.1	*Entamoeba histolytica*	MK332025.1	100
IQ-Human No. 6	OM268858.1	*Entamoeba histolytica*	MK332025.1	99.12
IQ-Human No. 7	OM268859.1	*Entamoeba dispar*	MK559465.1	100
IQ-Human No. 8	OM268860.1	*Entamoeba histolytica*	MK332025.1	99.13
IQ-Human No. 9	OM268861.1	*Entamoeba histolytica*	MK332025.1	99.09
IQ-Human No. 10	OM268862.1	*Entamoeba histolytica*	MK332025.1	100

NCBI-BLAST=National Center for Biotechnology Information-Basic Local Alignment Search Tool

The infection rate in cattle was 58%, as indicated by 58 positive samples from 100 tested. The percentage for age groups <1–6 months (77.14%) and 6–12 months (66.66%) was significantly higher than that for age groups 1–3 years (28.12%), as shown in Table-4 (p < 0.05). The infection rate was significantly higher in males (79.54%) than in females (41.07%) (p < 0.05; Table-5).

**Table-4:** Total infection rates of *Entamoeba* spp. in cattle according to age groups by conventional PCR.

Age/years	No. of samples examined	No. of positive samples	Percentage
<1–6 month	35	27	77.14
6–12 months	33	22	66.66
1–3 years >	32	9	28.12
Total	100	58	58
Chi-square 18.007, p = *0 (HS)			

* HS=Highly significant difference at p < 0.05. PCR=Polymerase chain reaction

**Table-5:** Total infection rates of *Entamoeba* spp. in cattle according to gender by conventional PCR.

Sex	No. of samples examined	No. of positive samples	Percentage
Females	56	23	41.07
Males	44	35	79.54
Total	100	58	58
Chi-square 14.97, p = *0 (HS)			

* HS=Highly significant difference at p < 0.05. PCR=Polymerase chain reaction

**Figure-4:**
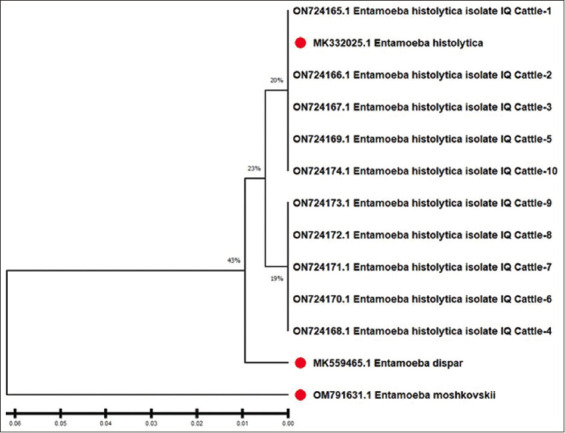
Isolated local cattle *Entamoeba* spp. was employed in phylogenetic tree analysis dependent on the partial sequencing of the 18S ribosomal RNA gene using genomic species typing analysis. Unweighted Pair Group Methods with Arithmetic Mean (UPGMA tree) were used to construct the phylogenetic tree (MEGA 6.0 version). 1–10 were identified as being closely linked to the *E. histolytica* (MK332025.1) strain by NCBI-BLAST, with total genomic alterations of 0.06%–0.01%.

DNA sequence in cattle 18S rRNA gene sequences were obtained from NCBI-GenBank for both *Entamoeba* species and their related strains, and DNA sequencing was conducted for genomic species type analysis. *E. histolytica* (MK332025.1) was the closest phylogenetic match to the genomic relationships of the isolated *Entamoeba* spp. cattle (No. 1–No. 10). The genomic identity of isolated cattle *Entamoeba* spp. ranged from 99.09% to 100%. 1–10, plus NCBI-GenBank entry MK332025.1, corresponds to Ent NCBI GenBank verified the submission of isolated cattle *Entamoeba* spp. using the accessions numbers ON724165.1 to ON724174.1 (Figure-4 and Table-6).

**Table-6:** The NCBI-BLAST homologous sequencing identity percentage between isolated local cattle *Entamoeba* spp. and NCBI-BLAST closed genomic relationship the isolated *Entamoeba* species.

*Entamoeba* spp. isolate	Accession number	Homology sequencing identity (%)		

		Identical *Entamoeba*	Accession number	Identity (%)
IQ-Cattle No. 1	ON724165.1	*Entamoeba histolytica*	IQ-Cattle No. 1	ON724165.1
IQ-Cattle No. 2	ON724166.1	*Entamoeba histolytica*	IQ – Cattle No. 2	ON724166.1
IQ-Cattle No. 3	ON724167.1	*Entamoeba histolytica*	IQ – Cattle No. 3	ON724167.1
IQ-Cattle No. 4	ON724168.1	*Entamoeba histolytica*	IQ- Cattle No. 4	ON724168.1
IQ-Cattle No. 5	ON724169.1	*Entamoeba histolytica*	IQ- Cattle No. 5	ON724169.1
IQ-Cattle No. 6	ON724170.1	*Entamoeba histolytica*	IQ- Cattle No. 6	ON724170.1
IQ-Cattle No. 7	ON724171.1	*Entamoeba Histolytica*	IQ- Cattle No. 7	ON724171.1
IQ-Cattle No. 8	ON724172.1	*Entamoeba histolytica*	IQ – Cattle No. 8	ON724172.1
IQ-Cattle No. 9	ON724173.1	*Entamoeba histolytica*	IQ- Cattle No. 9	ON724173.1
IQ-Cattle No. 10	ON724174.1	*Entamoeba histolytica*	IQ- Cattle No. 10	ON724174.1

NCBI-BLAST=National Center for Biotechnology Information-Basic Local Alignment Search Tool

## Discussion

The rate of *Entamoeba* spp. infection in humans, as determined by PCR, was 38%, which is higher than 9.3% in Salah Al-Din, Iraq [17] and 12% in Iran [18]. These differences may be due to suppression of the immune system and inadequate personal hygiene measurements, bad environmental conditions, retardation due to the lack of toilet management, direct contact from person to person [11], overcrowding, socioeconomic conditions, and malnutrition. In our study, age group of <10 years had the highest rate compared to the lowest in age group of 30–40 years. Barakat [19] reported the highest rate in age group of 1–30 years which was 78.79%, while Al-Hilfi *et al*. [11] reported the lowest rate in age group of 60–90 years which was 2.02% with a significant difference at p < 0.05. The highest infection rate in age group of 1–10 years may be due to this age spending more of their time outdoors playing, eating, discarded food, and staying put on the street and foraging in garbage dumps, touching with sands, and eating with dirty hands [20]. The school children involved in this study living in poor houses constructed with poor quality materials and no drainage [21]. Mahmood and Mustafa [9] reported the highest rate in age group of 36–45 years (8.3%), while Bahrami *et al*. [22] observed the maximum rate in age group of 30–50 years (28%). Another study recorded a low rate in age of 10–20 years and this may be due to young ages becoming more sanitation and hygienic associated with their looks, compared to those of lower age groups and accelerated ability to avoid contact as possible, which lead to get infection [23] or maybe extension of using metronidazole [24], and albendazole is given to school children in the campaign of national deworming and has been recorded that single dose of albendazole (400 mg) decrease *E. histolytica* infection in more than 50% of children of 7–15 years [25].

### Total infection rate of *Entamoeba* spp. in humans according to gender by PCR

In ThiQar, females had a higher prevalence of *Entamoeba* than males, as previously reported by Mahmood and Mustafa [9] and Flaih *et al*. [10]. These studies are not agree with previous studies in Iran [2, 11, 22], which pointed the highest rate in males compared to females, and this may be attributed to weak immunity and exhibit to infection, these variations due to ecological and physiological factors and hormonal sex-specific behaviors, also variation in endocrine immune system and males sexually mature and more susceptible to disease due to the sex steroids of hormones alternative the genes and behaviors that control stimulation and resistant to disease [26].

### Total infection rate of *Entamoeba* spp. in cattle according to age groups by PCR

Many studies have been conducted in cattle to detect the prevalence of *Entamoeba* spp., which is in accordance with current results (58%), such as 57.41% [27] and 54% [28] of *E. histolytica* in cattle. A lower infection rate, 45.6% was detected by Al-Areeqi *et al*. [8]. These differences are due to farmers’ disregard for culture and health, lack of commitment to health standards in the development of farms and animal breeding, ignorance during the movement of animals or use of water-polluted feed diseases, and differences in the geography and temperature of the region [29], environmental conditions, sample sizes, and immunity.

A study conducted by Naguib *et al*. [30] agree with our findings, which reported 62.79% for the 6–12 month age group and 34.28% for the age group above 12 months, with significant differences (p < 0.05; According to Naguib *et al*. [30], this discovery is linked to the adult cattle’s physiological condition due to their adaptive immunity against past infections. The various factors, including sample size, age groups, living conditions, management, season, and collection location, can influence the infection rate.

### Total infection rate of *Entamoeba* spp. in cattle according to sex by PCR

The study showed that 62.5% of males and 46.15% of females had significant differences (p < 0.05), which is consistent with Al-Maliki [28]. Female mammals exhibit greater resistance to parasite infections than males due to differences in exposure and immunosuppressive properties of testosterone. Male livestock carried higher parasite loads than their female counterparts.

### Phylogenetic analyses and sequencing

The current results are in agreement with many studies that found that *E. histolytica* was higher than *E. dispar* as 6% and 4.3% [9], 31.3% and 17.5% [10]; 66.03% and 11.32% in humans [28]. The number of patient samples assessed in the study and the time of specimen collection are contributing factors to the disparity in *E. histolytica* infection rates. The parasite may be acquired through ingestion of fecal matter from dogs, cats, sheep, and rodents. Both high temperatures and water contamination significantly contribute to the spread and completion of this parasite’s life cycle. Another factor is one’s economic standing.

Khan *et al*. [31] reported a different prevalence, *E. dispar* had higher prevalence (57.5%) than *E. histolytica* (47.5%). Another study reported a prevalence of 10% with *E. dispar* and 5% with *E. histolytica* in humans [11]. Like Alarady and Jasim [14], studies have reported *E. diaper* to be present in cows and sheep at respective levels of 35.7% and 21.45%, and *E. histolytica* at 85.7% and 21.4%. Environmental conditions, hygiene practices, population density, and DNA extraction from stool samples could account for observed differences.

In areas with extremely low standards of living and poor sanitation, *E. histolytica* thrives. The inadequate sanitary system may contribute to the high prevalence rate of *E. histolytica*. Contracting *E. histolytica* infections can be increased by keeping pets due to the parasite’s prevalence in animals and its spread through contamination. Therefore, it is proposed that more research should be conducted to identify and determine the genetic diversity of these parasites, as well as to determine the true pathogenicity and risk factors associated with *Entamoeba* species [31].

PCR-positive samples were identified as *E. histolytica* subtype 1 by sequence alignment (99% similarity) with accession number KB823016 [2].

According to the results of the BLAST analysis, six *E. dispar* amplicons (KY823418-KY823423), are 100% identical in sequence to accession number KP722600.1 in GenBank for *E. dispar*. The sequences KY823424 to KY823427 and KY884295 were identical to KP233840.1 (99%–100% homology). The phylogenetic analysis of *E. histolytica* gene sequences suggested that Iraqi isolates [22] are more closely related to Japanese isolates (AB282660.1 and AB485592.1) than to isolates from other countries. Accession numbers KT253450, KT253451, KT253452, KT253453, and KT253454 correspond to distinct isolates of a novel strain [12].

Phylogenetic sequence alignment of the local Iraqi isolate of *E. histolytica* revealed 100% identity with strain KF429800.1, which is more similar to *E. histolytica* than other *Entamoeba* species.

About 100% of the *E. histolytica* Iraq local isolates were identified as distinct from other *E. histolytica* isolates from AB282660.1 (Japan), KJ870211 (Cameroon), Y11272.1 (India), and GQ423749.1 (Philippines).

The five *E. histolytica* isolates from Iraq shared 98% identity with *E. dispar* (AB282661.1) and 100% identity with *E. histolytica* (AB282660.1 Japan); yet, significant genetic diversity exists among parasites exhibiting different morphologies. Morphological differences among *Entamoeba* species may not impact species-level variation, as some species infect multiple hosts [12].

## Conclusion

Phylogenetic analysis identified eight human isolates as *E. histolytica*, two as *E. dispar*, and 10 from cattle as *E. histolytica*. The high infection rate of *E. histolytica* in cattle compared to humans implies that the predominant species of this parasite in cattle significantly contributes to the transmission of this disease to humans and poses significant public health concerns.

## Authors’ Contributions

SMKA, HHA, and EJA: Conceptualization and data duration. SMKA: Formal analyses and drafted and revised the manuscript. HHA and EJA: Methodology. All authors have read, reviewed, and approved the final manuscript.
